# Impact of Opioid and Nonopioid Drugs on Postsurgical Pain Management in the Rat

**DOI:** 10.1155/2016/8364762

**Published:** 2016-03-16

**Authors:** Natalie M. Wilson, Matthew S. Ripsch, Fletcher A. White

**Affiliations:** ^1^Loyola University Chicago, Maywood, IL 60153, USA; ^2^Anesthesia, Indiana University School of Medicine, Indianapolis, IN 46202, USA; ^3^Richard L. Roudebush Veterans Administration Medical Center, Indianapolis, IN 46202, USA

## Abstract

*Aim*. Nonsteroidal anti-inflammatory drugs or opioids are commonly used to control surgical pain following veterinary and clinical procedures. This study evaluated the efficacy of postoperative ketorolac or buprenorphine following abdominal surgery.* Main Methods*. Mean arterial pressure (MAP), heart rate, animal activity, corticosterone levels, and a nociceptive sensitivity assay were used to evaluate 18 adult male Sprague-Dawley rats which underwent aortic artery occlusion for implantation of a radiotelemetry device. The animals were treated postoperatively with intraperitoneal injections of vehicle, ketorolac (10 mg/kg), or buprenorphine (0.06 mg/kg) every 8 hours for 3 days.* Key Findings.* There were no consistent significant changes in any of the telemetry parameters after treatment with ketorolac compared with no saline treatment with the exception of increased MAP in the buprenorphine group during the first 48 hours when compared with other treatment groups. There was a sustained increase in fecal corticosterone levels from baseline on days 2–7 with buprenorphine compared with vehicle- or ketorolac-treated animals. All treatment conditions displayed reduced paw withdrawal thresholds (PWTs) from day 1 to day 21 following surgery. Compared with the vehicle treatment group, buprenorphine-treated animals exhibited significantly lower PWT levels from day 4 to 14 days.* Significance*. Given the prolonged increase in fecal corticosterone levels and pronounced changes in tactile hyperalgesia behavior in rodents subjected to buprenorphine treatment, these data suggest that ketorolac may be superior to buprenorphine for the treatment of postprocedure pain behavior in rodents.

## 1. Introduction

The effective relief of postoperative pain is of paramount importance to the human or animal patient and ineffective pain relief following surgery may associate with the onset of chronic pain syndromes. More importantly for basic investigators, poorly controlled persisting pain following survival surgeries may both adversely impact the welfare of laboratory animals and confound the interpretation of experimental results.

The goal of proper postoperative pain management is to minimize pain and discomfort without producing unacceptable side effects. Nonsteroidal anti-inflammatory drugs (NSAIDs) such as ketorolac and/or opiate analgesics are traditionally used to control pain under these circumstances. Ketorolac is a potent analgesic commonly used for short-term management of postoperative pain [1]. Ketorolac, like other NSAIDs, has both analgesic and anti-inflammatory actions, with its mechanism of action predominantly through inhibition of the enzyme cyclooxygenase, inhibiting prostaglandin synthesis [2]. Side effects of chronic administration of NSAIDs include ulceration of the GI tract, impairment of platelet aggregation, nephrotoxicity, bone healing impairment, and hepatotoxicity. However, these problematic side effects are rarely seen with short-term administration [3, 4].

Severe pain arising from deep or visceral structures typically requires the use of opiates. The effective duration of many opiates is short in rodents, making several of these compounds (morphine, meperidine, and pentazocine) impractical. A widely used opiate in rodents and companion animals is buprenorphine, a mixed agonist and antagonist with persisting clinical effects (8–12 hours) [5]. However, buprenorphine shares many side effects with other opiates. The most common side effects in rodents include constipation and drowsiness. Larger doses produce respiratory depression and hypotension.

Assessing the level of pain in rodents recovering from surgery is controversial. General indicators of pain include decreased water and food intake, loss of weight, decreased home cage activity, and altered social interactions with others in the cage [6–8]. Other indicators of localized discomfort include spontaneous biting of affected limbs, flinching, guarding, licking, lifting, and shaking [9]. Additional methods for assessing pain in rodents include the measurement of involuntary movements made in response to a stimulus. For example, the nociceptive withdrawal reflex to a high-intensity stimulus is thought to reflect acute pain while the presence of spontaneous or evoked behavioral responses in animals may be indicative of a persistent pain. It has also been suggested that changes in heart rate, respiratory rate, and blood pressure and changes in the hypothalamus-pituitary-adrenal (HPA) axis may provide information on surgical pain states in rodents [10–12].

The current experiments were conducted using rats subjected to abdominal surgery. The telemetry devices recorded the effects of postoperative treatment with vehicle, ketorolac, or buprenorphine on blood pressure, heart rate, and home cage activity. We also tested the hypothalamus-pituitary-adrenal (HPA) axis function as measured by fecal corticosterone levels and determined the presence of tactile hyperalgesia as assayed with von Frey-type probes in conscious, unrestrained rodents. A major result of this rodent study indicates that ketorolac during the postoperative period provides superior analgesic control to buprenorphine over a period of 21 days.

## 2. Methods

### 2.1. Animals

Pathogen-free, adult male Sprague-Dawley rats (250–300 g; Harlan Laboratories, Madison, WI) were housed in constant temperature (23 ± 3°C) rooms with standard rodent chow and water available ad libitum. The animals were subjected to consistent cycles of light and darkness (12 h light : 12 h dark cycle, lights on at 07:00 h and off at 19:00 h). Experiments were performed during the light cycle. All animals were subjected to behavioral assays prior to treatment and randomly assigned to one of the three experimental treatment groups (vehicle *n* = 7, ketorolac *n* = 8, and buprenorphine *n* = 8). These experiments were approved by the Institutional Animal Care and Use Committee of Loyola University, Chicago. All procedures were conducted in accordance with the Guide for Care and Use of Laboratory Animals published by the National Institutes of Health and the ethical guidelines of the International Association for the Study of Pain. All behavioral testing of the treatment groups took place within the Loyola University Animal Facility.

### 2.2. Telemetric Studies for Analysis of Blood Pressure, Heart Rate, and Activity in Conscious Unrestrained Rats

While under surgical levels of isoflurane anesthesia (as defined by lack of purposeful response to surgical incision), biotelemetry blood pressure/activity transmitters (Model TA11PA-C40; Data Sciences, St. Paul) were implanted in the abdominal cavity of the rats. The nonflushable catheters for measuring arterial pressure were inserted into the descending aorta just below the renal artery following the permanent occlusion of the abdominal aorta 10 mm below renal artery bifurcation. Signals arising from each transmitter were captured by a telemetry receiver interfaced with a microcomputer for data acquisition and analysis. All surgeries were performed between 8 AM and noon, and values for heart rate, blood pressure, and activity were recorded continually at a 10-minute interval beginning at 2 PM on the day of implantation. Data were used to examine average hourly values for heart rate, mean arterial pressure (MAP), and activity beginning after recovery from surgical anesthesia and demonstration of normal conscious behaviors. The activity count is measured as a change in the signal strength from the transmitter/telemetry device and the receiver, which can occur from a change in the distance or orientation. This count does not represent a measure of distance traveled. Therefore, activity is presented as a relative measure of animal movement around the cage.

### 2.3. Analgesics

Drugs utilized in this study represent the rat formulary doses suggested by NIH Office of Animal Care and Use (http://oacu.od.nih.gov/arac/documents/Pain_and_Distress.pdf) and were administered immediately after completion of surgery and every 8 hours thereafter for a total of 9 doses (ketorolac, 10 mg/kg, intraperitoneal; buprenorphine, 0.06 mg/kg, intraperitoneal; or vehicle/saline, intraperitoneal). The last dose was administered 64 hours after completion of surgery.

### 2.4. Fecal Corticosterone

All animals were housed individually in cages before and after implantation of the telemetry devices. Fecal pellets were collected on the morning before the implantation surgery and each morning for days 1–3 and then every other day for 13 days; the pellets were frozen at −20°F until assayed. On the day before the assay, each pellet sample was ground down to a powder and then weighed for an amount between 0.45 and 0.55 grams and placed into individual sample tubes. Next, 5 mL of ethanol was added to each tube; tubes were rocked overnight to extract corticosterone. Following centrifugation at 1500 rpm for 15 minutes, the corticosterone concentration in the supernatant was determined utilizing a corticosterone enzyme immunoassay kit (Assay Designs, Ann Arbor, MI) according to the manufacturer's instructions. Values were reported as ng corticosterone/gm fecal pellet.

### 2.5. Foot Withdrawal to Punctate Mechanical Indentation

Foot withdrawal in response to mechanical indentation of the plantar surface of each hind paw was assessed with von Frey-type filaments. Mechanical stimuli were applied with seven filaments, each differing in the bending force delivered (10, 20, 40, 60, 80, 100, and 120 mN). Each filament was fitted with a flat tip and a fixed diameter of 0.2 mm [13–16]. The force equivalence of mN to grams is as follows: 100 mN equals 10.197 g.

Rats were placed on a metal mesh floor and covered with a transparent plastic dome. Typically, the animals rest quietly in this situation after an initial period of exploration. Animals were habituated to this testing apparatus, two days prior to the behavioral assays. Following acclimation, each filament was applied to six locations spaced across the nerve distribution of hind paw glabrous skin. The filaments were tested in order of ascending force, with each filament delivered in sequence from the 1st to the 6th location alternating from one hind paw to the other. The duration of each stimulus was 1 second and the interstimulus interval was 10–15 seconds. A cutoff value of 120 mN was used; animals that did not respond at 120 mN were assigned that value [15, 16]. In each behavioral testing sequence, the operator was blinded to the animal treatment condition and the blinding codes were not revealed until the completion of the data collection.

The incidence of paw withdrawal was expressed as a percentage of the six applications of each filament as a function of force. A Hill equation (coefficient of 1) was fitted to the function (Origin version 6.0, Microcal Software, Northampton, MA) relating the percentage of indentations eliciting a withdrawal to the force of indentation. From this equation, the paw withdrawal threshold (PWT) force was obtained and defined as the force corresponding to a 50% withdrawal. At least a −20 mN difference from the baseline PWT in a given animal is representative of mechanical hypernociception [15, 16]. The experimenter was blinded to both the injury condition of the animal and the drugs utilized in all behavioral trials.

### 2.6. Statistics

Biotelemetric activity and fecal cortisol values were evaluated using ANOVA with post hoc analysis (SigmaStat, SPSS, Inc.). The criterion for statistical significance was *p* < 0.05. Values are presented as mean ± SEM. Statistical analysis of changes in mechanical paw withdrawal thresholds was carried out using repeated measures two-way ANOVA followed by Bonferroni's post hoc test. The behavioral criterion for statistical significance was *p* < 0.05.

## 3. Results

### 3.1. Heart Rate

**Figure 1:**
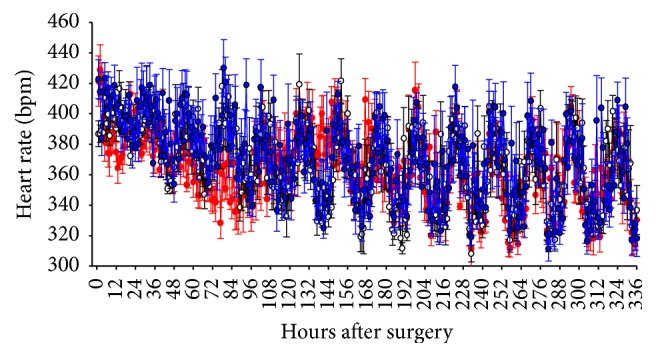
Heart rate is not altered by surgical procedure in combination with vehicle, ketorolac, or buprenorphine. Heart rate in saline-treated (*n* = 7, open circles), ketorolac-treated (*n* = 8, blue closed squares), and buprenorphine-treated (*n* = 8, red closed circles) rats after surgery for implantation of biotelemetry devices. Treatment with analgesic or saline was continued for three days, with the last dose being given 64 hours after surgery. Time 0 represents 2 PM on the day of surgery, a time at which all animals had recovered from anesthesia.

Opiates have little effect on the myocardium during surgical procedures; however postoperative monitoring of heart rate indicates that there was a trend towards a lower heart rate in the buprenorphine-treated rodent as compared to the saline and ketorolac groups during the first 4 days after surgery (Figure 1) though these differences were not statistically significant. Heart rate was significantly greater in the buprenorphine group as compared to the other two groups at most intervals from postoperative hours 140 through 146 (76 hours after the last dose of buprenorphine). No other time- or group-related differences were observed in heart rate.

### 3.2. Mean Arterial Pressure (MAP)

**Figure 2:**
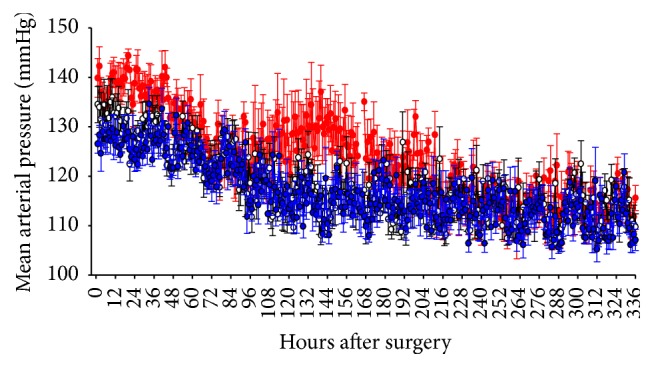
Mean arterial pressure is not altered by surgical procedure in combination with vehicle, ketorolac, or buprenorphine. Mean arterial pressure in saline-treated (*n* = 7, open circles), ketorolac-treated (*n* = 8, blue closed squares), and buprenorphine-treated (*n* = 8, red closed circles) rats after surgery for implantation of biotelemetry devices. Treatment with analgesic or saline was continued for three days, with the last dose being given 64 hours after surgery. Time 0 represents 2 PM on the day of surgery, a time at which all animals had recovered from anesthesia.

Normal values for average blood pressure during a cardiac cycle during surgical procedures vary with species and the cardiovascular action of opiates may further alter normal physiologic values. As shown in Figure 2, there were no differences in MAP between saline-treated and ketorolac-treated rats at any time after implantation of the telemetry devices. Values for MAP in buprenorphine-treated animals were significantly greater than those in the other two groups during the first two days after surgery and again on days 5 through 7; values in the buprenorphine group were also significantly greater at several sample periods between postoperative hours 11 and 30 and postoperative hours 123 and 160.

### 3.3. Home Cage Activity

**Figure 3:**
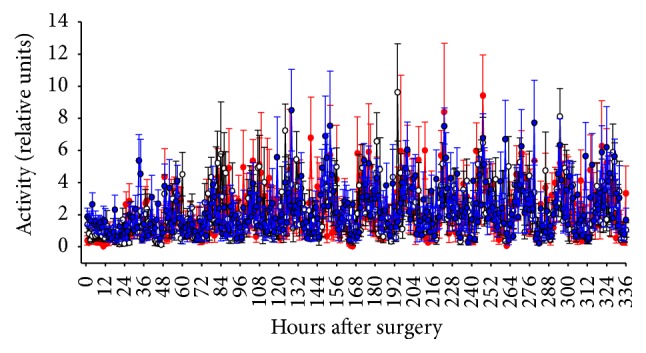
Home cage activity is not altered by surgical procedure in combination with vehicle, ketorolac, or buprenorphine. Activity in vehicle-treated (*n* = 7, open circles), ketorolac-treated (*n* = 8, blue closed squares), and buprenorphine-treated (*n* = 8, red closed circles) rats after surgery for implantation of biotelemetry devices. Treatment with analgesic or saline was continued for three days, with the last dose being given 64 hours after surgery. Time 0 represents 2 PM on the day of surgery, a time at which all animals had recovered from anesthesia.

Uninterrupted recordings of home cage activity were obtained in all three treatment conditions. As shown in Figure 3, animal movement activity did not differ among the three treatment groups at any time after abdominal surgery.

### 3.4. Fecal Corticosterone

**Figure 4:**
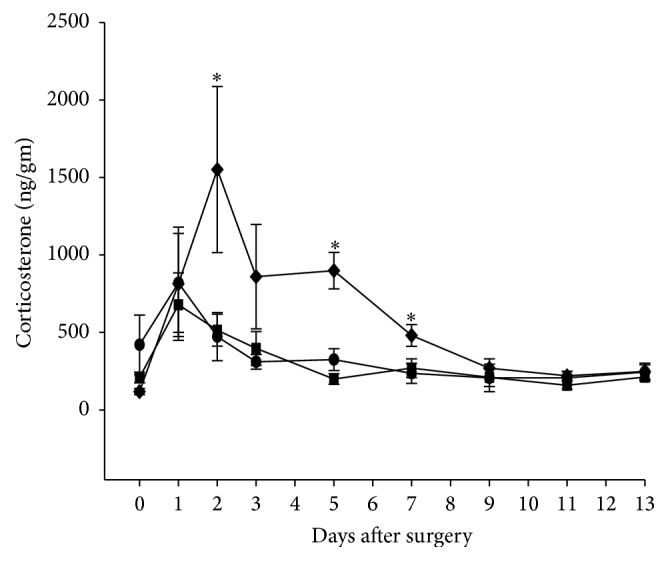
Fecal corticosterone levels in vehicle-treated (*n* = 7, closed circles), ketorolac-treated (*n* = 8, closed squares), and buprenorphine-treated (*n* = 8, closed diamonds) rats before and after surgery for implantation of biotelemetry devices. Treatment with analgesic or saline began immediately after surgery and was continued for 3 days. Fecal pellets were collected on days 2, 3, 5, 7, 9, 11, and 13 between 8 AM and noon. Significantly different values were collected for only the buprenorphine-treated group when compared with saline- and ketorolac-treated animals; ^*∗*^ *p* < 0.05.

Corticosterone is a stress hormone that is regulated through activation of the HPA axis. Stress and injury have been shown to lead to increased circulating levels of corticosterone [12, 17]. Fecal samples represent effective and noninvasive means to measure changes in corticosterone [18–22]. Therefore, to determine the degree to which abdominal surgery in combination with saline, ketorolac, or buprenorphine contributes to a stress response, we measured fecal corticosterone for 13 days. As shown in Figure 4, there were no significant differences in fecal corticosterone levels among the three groups of animals prior to surgery (day 0). Values in each group were increased above control levels on the first day after surgery (day 1). In the saline and ketorolac groups, values reached their greatest levels on day 1 and returned to near control values by day 3 after surgery, remaining unchanged during the remainder of the 13-day period. In contrast, values in the buprenorphine group continued to increase on day 2 after surgery and recovered gradually back to control levels by day 9. Values in the buprenorphine group were significantly greater than those of the other two groups on days 2, 5, and 7.

### 3.5. Injury-Induced Alterations in Tactile Hyperalgesia

**Figure 5:**
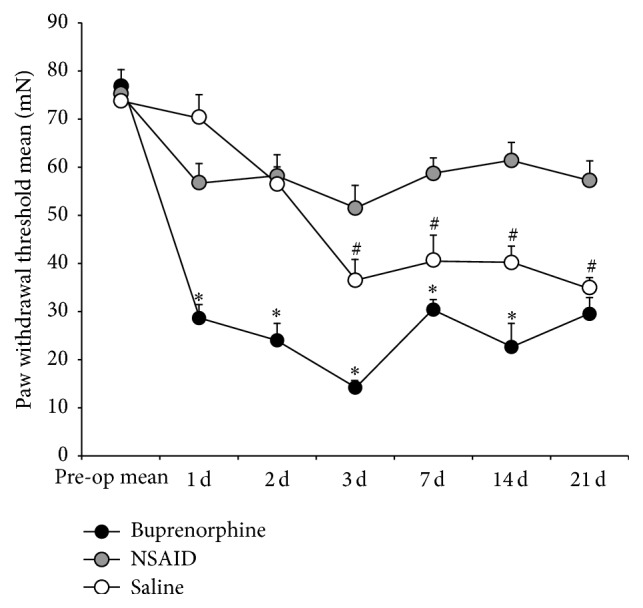
Mean mN change in paw withdrawal threshold of the vehicle-treated group (*n* = 7), buprenorphine-treated group (*n* = 8), and ketorolac-treated group (*n* = 8). Vehicle treatment group was significantly decreased by the end of postsurgery day 1 through day 21. Treatment groups comparison demonstrated that the buprenorphine group exhibited paw withdrawal thresholds significantly lower than vehicle-treated groups from postsurgery day 1 through day 14 (^*∗*^ *p* < 0.001). Ketorolac treatment induces a significant difference in PWT when compared to vehicle-treated animals from postsurgery day 3 through day 21 (^#^ *p* < 0.001). Error bars represent SEM.

The effect of abdominal surgery in combination with vehicle, ketorolac, and buprenorphine on reflexive withdrawal to mechanical stimulus was assessed using von Frey filaments. Separate groups of rats were used over a course of 21 days. Prior to surgery, the average PWT response to the von Frey test was 75 ± 3 mN (Figure 5). From day 1 to day 21, all treatment groups PWTs were significantly different from presurgical baseline levels. However, vehicle-group-versus-treatment-group differences were limited to only the buprenorphine-treated animals on day 1. The buprenorphine-treated animals were significantly lower than the vehicle-treated animal responses (buprenorphine, 24 ± 3.77 mN versus vehicle, 56.5 ± 3.5; repeated measures ANOVA* F* = 24.54, *p* < 0.0001). Statistically significant differences between buprenorphine and vehicle-treated animals groups continued through day 14. The NSAID-treated group did not differ from vehicle-treated animals until day 4. On day 4, NSAID-treated animals exhibited a PWT of 51.5 ± 4.67 which was significantly higher than vehicle-treated PWT of 36.5 ± 4.5 (repeated measures ANOVA* F* = 23.99, *p* < 0.0001). Statistically significant differences between NSAID- and vehicle-treated animals groups continued through day 21.

## 4. Discussion

Ketorolac and buprenorphine were studied in rodents subjected to abdominal placement of biotelemetry blood pressure/activity transmitters in an effort to identify which drug treatment provided better control of postsurgical physiological changes, corticosterone levels, and pain behavior in rodents when compared to vehicle. With the exception of a transient change in MAP four days after surgical procedure in the animals subjected to buprenorphine, biotelemetry changes were not altered in the rodents. Fecal corticosterone levels were elevated for saline and ketorolac treatments during the first 24 hours but returned to baseline for the remainder of the study. In contrast, buprenorphine-treated animals exhibited elevated corticosterone levels for six days when compared with saline or ketorolac treatments. Moreover, behavioral analysis of the treatment conditions revealed that postsurgical buprenorphine treatment significantly* enhanced* the display of tactile hyperalgesia from day 1 to day 21 when compared with the ketorolac-treated group.

Ketorolac is a frequently used nonsteroidal anti-inflammatory drug to treat postoperative pain. The mechanistic actions of ketorolac include inhibiting the bodily synthesis of prostaglandins including prostaglandin E2 (PGE2). The nonselective inhibitory effects of ketorolac on cyclooxygenase products associated with PGE2 production due to tissue damage likely provide much of the peripheral analgesia observed with exposure to the drug treatment [23]. The degree to which the drug contributes to central nervous system effects is unknown though biodisposition of ketorolac within the central nervous system may contribute to greater behavioral efficacy for tactile allodynia as opposed to modulation of thermal nociception [24, 25]. Despite the apparent advantages of both selective and nonselective COX inhibitor to provide analgesia in perioperative settings, long-term use of these drugs is limited due to a number of off-target effects including effects on the cardiovascular system, gastrointestinal erosions, and renal and hepatic insufficiency [26].

Opioid drugs, such as buprenorphine, are important components of many surgical anesthesia regimens and widely believed to be the most potent available postprocedural analgesics. Buprenorphine is often used as it is longer-acting and is good for most postoperative applications. The duration of action is thought to be 6–12 hours; however it is closer to 6 hours in most situations. As noted in Methods, the recommended dose of buprenorphine utilized by the NIH Office of Animal Care and Use (OACU) for rat laparotomy is 0.01–0.05 mg/kg SC or IP. However, as this laparotomy procedure produces a permanent occlusion of the abdominal aorta, we opted to use the 0.06 mg/kg in these studies [27]. Despite the increased dose, buprenorphine was less effective than ketorolac in our behavioral studies. Other groups have noted that this commonly accepted “gold standard” opioid was also not effective for postoperative analgesia in male or female Long-Evans or Sprague-Dawley rats [28–30]. One interpretation of the observed enhancement of pain-like behavior in the buprenorphine-treated animals for up to three weeks after the surgical procedure may be due to a buprenorphine-induced change in the behavioral state [31]. To this end, there are a growing number of publications that provide evidence that opiate analgesics (e.g., morphine, fentanyl, hydroxymorphine, methadone, and perhaps buprenorphine), in addition to their antinociceptive properties, can also activate a pain facilitatory system that effectively enhances pain-like behavior in preclinical conditions [32–37] and pain sensitivity in clinical situations [38–40]. This condition is commonly referred to as opiate-induced hyperalgesia (OIH).

The molecular mechanisms of OIH are largely unknown although OIH appears to share characteristics of neuropathic pain and consequently has important clinical implications and can be defined as an increasing sensitivity to noxious stimuli, even evolving a painful response to previously nonnoxious stimuli. Some groups have suggested that OIH may be due to tonic, descending facilitation in the dorsal horn of the spinal cord through changes in activity of NMDA receptors [41]. Although neither of these modes of action can be completely ruled out, the clinical OIH mechanism may also depend on opioid-induced activation of neuronal Toll-like receptor 4 (TLR4) [42]. Recent preclinical evidence suggests that opioid activation of TLR4 present on nociceptive primary sensory neurons cells serves to increase the voltage gated sodium channel (VGSC-NaV1.7; SCN9A in humans) current density which contributes directly to the diminished efficacy of opioids in chronic pain management [36, 43].

Corticosterone levels were monitored using fecal corticosterone samples. Plasma corticosterone has previously been the preferred method for monitoring corticosterone. However, fecal corticosterone measurements allow for a noninvasive means to monitor corticosterone levels and can detect up to 80 percent of systemically administered radioactive corticosterone [18, 19]. The greatest advantage to utilization of fecal samples is the lack of handling or restraint required, which have been shown to produce increases in circulating corticosterone levels [20–22, 44].

The observed prolonged increase in fecal corticosterone levels following buprenorphine injections represents an interesting insight into the state of these animals after surgery. Although the animals across the treatment groups exhibited similar activity levels the corticosterone levels were elevated only in the buprenorphine treatment group. Glucocorticoids such as corticosterone are known as stress hormones whose regulation is controlled by the HPA axis. Stimulation of the HPA axis through such effectors as stress or injury can lead to increased circulating levels [12, 17]. Additionally, these enhanced circulating levels have been shown to contribute to nociceptive behavior, which can be reversed by the glucocorticoid receptor antagonist, RU486 [22]. Furthermore, opioid treatment previously has been shown to increase circulating corticosterone levels in a number of studies through activating the HPA [45–47], whereas the use of NSAIDs does not lead to an increase in corticosterone levels [48, 49].

The presence of tactile hyperalgesic behavior following surgical procedures is not a novel finding in postsurgical conditions [23]. However, given that placement of the telemetry device and the resultant occlusion of the abdominal aorta permanently impact vascular perfusion of the lumbosacral spinal cord as well as the ancillary sensory ganglion and affiliated peripheral nerves the production of prostaglandins and proinflammatory cytokines and chemokines in the periphery may contribute to the maintenance of this type of tactile hyperalgesia beyond the testing period [50].

In summary, our results show that ketorolac is superior to buprenorphine in rats recovering from abdominal surgery. Buprenorphine enhanced the tactile hyperalgesic behavior following surgery and was associated with increased fecal corticosterone levels. These data indicate that standard opioid treatment of postsurgical pain may not lead to the best outcomes in rodents subjected to major survival surgical procedures.
